# Social skills in future dental students – a project report

**DOI:** 10.3205/000346

**Published:** 2025-09-23

**Authors:** Johanna Hissbach, Sinikka Heisler, Oana Gröne, Stefanie Pfisterer-Heise

**Affiliations:** 1 University Medical Center Hamburg-Eppendorf, Germany; 2 German Sport University Cologne, Germany

**Keywords:** social competence, dentistry, student selection, interviews, Situational Judgment Test

## Abstract

**Objective:**

The doctor-patient interaction is essential for successfull dental treatment. Although it is possible to consider social skills during student selection, these are rarely taken into account. The described project aims to identify and evaluate the social skills deemed necessary by various stakeholders and to assess whether these skills can be effectively measured using a Situational Judgment Test (SJT).

**Methods:**

The project involved conducting interviews with stakeholders (lecturers, students, patients, practicing dentists) to identify relevant social skills. This was followed by a Delphi survey to evaluate the importance of these skills. Additionally, the SJT was examined for its suitability in the context of dental medicine, and various methods for reliably measuring the identified skills were assessed.

**Results:**

Dental lecturers and students consider emotional resilience, particularly stress management, to be especially important during dental studies, while patient-related behaviors are of lesser priority – possibly due to the constraints of the academic environment. In contrast, patients and dentists emphasize the importance of helpfulness and caring conduct during treatment.

**Conclusion:**

Our research highlights the need to strengthen social skills in dental education. Although the SJT from general medicine is also suitable for dental studies, Multiple Mini Interviews (MMIs) are a more effective method for capturing complex skills, such as behavioral flexibility.

## Zusammenfassung

**Zielsetzung:**

Die Arzt-Patienten-Interaktion ist entscheidend für den Therapieerfolg in der zahnmedizinischen Versorgung. Trotz der Möglichkeit, soziale Kompetenzen auch schon bei der Studierendenauswahl einzubeziehen, werden diese bislang wenig berücksichtigt. Das beschriebene Projekt zielt darauf ab, die aus Sicht verschiedener Interessengruppen erforderlichen sozialen Kompetenzen zu ermitteln und zu bewerten, inwiefern sich diese für die Erfassung mittels eines Situational Judgement Tests (SJTs) eignen.

**Methoden:**

Im Projekt wurden Interviews mit Interessenvertreter*innen (Lehrenden, Studierenden, Patient*innen, niedergelassenen Zahnärzt*innen) zur Identifikation relevanter Kompetenzen geführt, gefolgt von einer Delphi-Befragung zur Bewertung ihrer Wichtigkeit. Darüber hinaus wurde der bestehende SJT auf seine Eignung für die Zahnmedizin untersucht und mögliche Methoden zur zuverlässigen Messung der identifizierten Kompetenzen evaluiert.

**Ergebnisse:**

Lehrende und Studierende der Zahnmedizin halten emotionale Resilienz, insbesondere Stressbewältigung, für das Studium für besonders wichtig, während patient*innenbezogene Verhaltensweisen weniger Priorität haben; dies könnte auf die Rahmenbedingungen im Studienalltag zurückzuführen sein. Im Gegensatz dazu betonen Patient*innen und Zahnärzt*innen die Bedeutung von Hilfsbereitschaft und Fürsorge in der Behandlung.

**Schlussfolgerung:**

Die Forschungsergebnisse zeigen die Notwendigkeit, soziale Kompetenzen im Zahnmedizinstudium zu stärken. Der SJT aus der Humanmedizin eignet sich zwar auch für die Zahnmedizin, Multiple Mini Interviews (MMIs) sind jedoch eine effektivere Methode zur Erfassung komplexer Fähigkeiten, wie z.B. Flexibilität im Verhalten.

## 1 Introduction

The dental care of the population plays an important societal function in social and economic terms [1]. At the same time, visiting the dentist is often associated with uncomfortable emotions for many people. In a survey, more than 25 percent of patients reported suffering from dental anxiety [2]. Against this background, knowledge and technical skills are not the only prerequisites for successful dental treatment [3]. In a survey, 95 percent of dentists stated that the dentist-patient relationship has a decisive influence on treatment success [4]. The perceived negative behavior of dentists plays a crucial role in the development of “dental anxiety” [5]. Accordingly, licensed dentists should possess strong social skills that enable them to treat the whole person, not just the mouth [6]. These include, for example, transparent communication, ethical conduct, or the ability to recognize and resolve conflicts [7]. These social skills support building a relationship with the patient and are associated with increased treatment satisfaction and improved treatment outcomes [7].

In response to this demand, the German National Competency-Based Learning Objectives Catalog in Dentistry (NKLZ), introduced in 2015 [1], aims to address it by serving as a graduate profile or professional qualification framework for dentists up to the point of licensure. Based on the Canadian CanMEDS framework [8], the learning objectives of dental studies are defined, including, for the first time, social and communicative skills. The definition of these social and communicative skills, to be learned during studies, was informed by the perspectives of medical didacticists and subject matter experts through a consensus process. However, it has not been systematically researched which social skills patients and dental students consider important for successful practice or the study of dentistry. Active involvement of patients in shaping educational content not only contributes to the development of empathetic and competent clinicians but also helps to align dental education more closely with the individual patients’ needs [9]. These additional perspectives can validate previous research or allow the identification of further, previously overlooked factors.

In Germany, social skills have so far received little attention as selection criteria for dental studies [10]. Following the 2017 German Federal Constitutional Court ruling [11], the entire admission system for medical degree programs was restructured. Just as manual skills are now included in applicant selection, social skills could also be taken into account. However, only two of the 29 dental faculties considered social skills in their applicant selection process for the winter semester starting 2024 [12].

Internationally, efforts have been made with varying success to incorporate criteria outside the academic realm [13], [14], [15], [16], [17], [18], [19]. A method to measure social skills is the so-called Situational Judgment Test (SJT), in which several scenario descriptions and related possible responses are presented. Depending on the test format, applicants may, for example, be asked to choose the best course of action or evaluate the effectiveness of various alternatives [20]. In a review of the effectiveness of various selection methods in human medical student admissions, Patterson et al. [21] concluded that SJTs are reliable, valid, and cost-efficient.

Few studies have so far addressed SJTs for selecting dental students. In Belgium, Buyse and Lievens [22] reported a weak but statistically significant correlation between results of a video-based SJT and performance in the fifth year of study, which might be explained by the increased practical relevance of this stage. Similarly, a study of the SJT component of the UK Clinical Aptitude Test (UKCAT) found a low correlation with the interview procedure of an English university [23]. Both SJTs were not specifically developed for dentistry and were thus used in both dentistry and general medicine.

Since 2016, various versions of SJTs have been used and scientifically evaluated at the University Medical Center Hamburg-Eppendorf (UKE) for applicant selection [24], [25], [26]. In the field of general medicine, pilot studies show satisfactory internal consistency and weak positive correlations with the HAM-Int interview method [27] as well as with Objective Structured Clinical Examination (OSCE) results [28]. It remains an open question to what extent an SJT developed for general medicine is suitable for assessing the social skills relevant to dentistry.

## 2 Project description

As a consequence of the Federal Constitutional Court ruling, the development of the NKLZ, and the introduction of the model degree program iMED DENT, the selection criteria for dental studies at the University Medical Center Hamburg-Eppendorf were expanded to include social compentences. The aim of this project was to advance research on social competences in dentistry, with particular attention to different interest-holders and the distinction between study and professional practice. Additionally, it sought to identify which methods are suitable for assessing the skills deemed important in an admissions process.

### 2.1 Research questions

1. Which social skills are required by future dentists for succeeding in dental studies and their subsequent professional practice, from the perspectives of various stakeholders?
2. Which of the identified social skills are most important for dental studies and professional practice?
3. To what extent is a Situational Judgment Test (SJT) used in human medicine a suitable method for assessing the relevant social skills, and what other assessment methods may be available within the selection process?

### 2.2 Project implementation

To address these research questions, two sub-projects were planned: “Exploration and Evaluation of Social Skills for Dental Studies and Professional Practice” and “Evaluation of the HAM-SJT for the Field of Dentistry”. Due to the COVID-19 pandemic, the original project plan had to be adjusted. In the first phase of the project, the planned focus groups could not take place; instead, expert interviews were conducted by phone.

For both the interview study and the Delphi study, data protection concepts were developed, and positive ethics approvals were obtained from the Local Psychological Ethics Committee at the Center for Psychosocial Medicine LPEK (interview study 0154, Delphi study 0221).

### 2.3 Objectives and methods

#### Exploration and evaluation of social skills: interview study (study 1)

Initially, an interview study was conducted with stakeholders in the field of dentistry (patients, practicing dentists, lecturers, students) to identify relevant social skills. The aim was to explore the different perspectives of stakeholders on the social skills necessary for dental students and practicing dentists. Participants were recruited through (1) postings in supermarkets in various districts of Hamburg, (2) distribution of flyers to randomly selected dental practices, (3) newsletters to UKE students, and (4) personal contacts, particularly for the age group of over 60-year-old patients. The aim of this purposive sampling was to capture a broad variability within the examined group. For this, qualitative sampling plans were developed for each sub-population, considering various factors and their variations (patients: dental anxiety, age, and number of dental visits per year over the past five years; practicing dentists: age, treatment of mainly statutory vs. private patients, migration background; lecturers: age, migration background; students: semester count, satisfaction with the course, migration background). In total, 27 individuals were interviewed, comprising ten patients (70% women), four dentists (50% women), four lecturers (25% women), and nine students (44% women). All participants received an Amazon voucher worth 25 euros as an incentive.

Based on the Critical Incident Technique [29], a semi-structured interview guide was developed, tailored to each subgroup (dentists, patients, students) and their everyday life experiences. The interview questions addressed participants’ ideas about socially competent dentists, their behavior in routine and challenging situations, and difficult or particularly positive experiences during dental treatments. To avoid influencing respondents’ thoughts and statements, no preset definitions of social skills were provided; instead, subjective definitions and perceptions of respondents were used.

All interview data were content-semantically transcribed [30] and analyzed using thematic analysis [31] with the MAXQDA 2020.4.2 software [32]. Initially, the transcripts were read and inductively coded. The codes were then grouped, and inductive themes were identified. In the third step, the identified themes were deductively mapped onto the social skills inventory by Kanning [33]. The concept of “competence” inherently involves the question of whether it refers to state- vs. trait-variables. Kanning [34] distinguishes between social competences and socially competent behavior.

#### Evaluation of social skills: Delphi study (study 2)

The codes (see Table 1 (Tab. 1)) derived from the interview study were used for the subsequent Delphi study, aiming to evaluate the importance of these themes for dental studies and professional practice.

A two-round Delphi survey was conducted using the online tool LimeSurvey 3.0.0 [35]. All interview participants from the telephone study who had provided consent for further contact were invited to participate. Additionally, the group of patients was expanded via a convenience sample to include persons over 60 years old. In total, a sample of 79 persons was recruited, consisting of 31 patients (65% women; mean age=42.8 years), 11 practicing dentists (82% women; mean age=36.1), 11 lecturers (55% women; mean age=39.3), and 26 students (88% women; mean age=24.0). They rated the identified themes, which included behaviors and skills, regarding their importance for dental studies and practice.

In the first Delphi round, *dentists and patients* were asked to select up to 25 of what they considered the most important competences and behaviors for *dental practice* (out of 49). Simultaneously, *lecturers and students* chose the competences and behaviors they deemed most relevant for *dental studies*. In the second round, both groups were presented with their results (the 25 most important competences and behaviors, plus equal ranked categories) and asked to reorder them according to their importance.

#### Assessment of the HAM-SJT for dentistry: matching skills with constructs in the current SJT for human medicine (study 3)

The 15 most important skills and behaviors for both study and professional practice were examined to evaluate their suitability for selection procedures prior to starting studies or for competency assessment after graduation, as well as to identify appropriate methods for measurement. The SJT items used in the 2020 human medicine selection process were initially mapped to social skills and then assessed for their suitability in dental student selection.

Two experienced experts in SJT development evaluated all SJT items used in the Hamburg medical student selection, focusing on: Which behaviors are prominent in the described situations? Do these competences and behaviors align with those relevant to dentistry?

#### Consensus rating on methods for measuring the identified skills (study 4)

The goal was to evaluate whether the identified behaviors and competences could be reliably measured using various methods (Multiple Mini Interviews (MMI), Situational Judgment Tests, personality questionnaires, Objective Structured Clinical Examination (OSCE)). The purpose was to determine which approaches are most suitable for accurately and reliably capturing these skills, thus forming a solid basis for selection procedures.

Two highly experienced staff members conducted a consensus rating, assigning appropriate measurement methods to the identified behaviors and competences. They evaluated which approaches are meaningful and effective for assessing each competence.

## 3 Results

### 3.1 Interview study

Overall, 49 codes emerged from the interviews, which were assigned to the 17 themes within Kanning’s framework [33] (see Table 1 (Tab. 1)). Although “social skills” were not explicitly defined in the interviews, aspects from all 17 Inventory of Social Competences (Inventar Sozialer Kompetenzen, ISK) scales were coded, with the theme “helpfulness/care”, linked to the “prosociality” ISK scale, being the most elaborated.

Two themes were identified that could not be placed within the framework: “professional ethics” and “self-organization”. Additionally, lecturers from UKE and practicing dentists highlighted further aspects not categorized under “social skills”, specifically factors related to dental clinical competence and professional attitudes. These included aspects such as business management (running a practice), manual dexterity and meticulousness in work, a structured approach to tasks, and physical prerequisites for practicing the profession (e.g., physical fitness, tolerance to noise). The surveyed dentists expressed concerns about inadequate preparation during their studies concerning interpersonal skills. Regarding dental treatment, they focused on specific features of the dental practice setting, particularly the patient’s physical position during treatment (quote: “[...] like how you have a submissive animal or something. I know that from my dogs and such, that even when they submit, they lie on their back and present their throat, and that’s how the patient naturally feels helpless. I think that also plays a part in the patient’s psyche, that they are there, and you need to try to resolve or not present yourself as the one [...] or someone who knows everything and stands above the patient.” 

Similarly, interviewed patients emphasized the importance of creating a “safe space” during dental treatment (quote: “Hm. Empathy. (...) I don’t know, feeling seen and taken seriously in the – whether with pain at the moment or not – hm. (...) Because it is a very intimate or close matter (...) hm, and appreciating that (...) it’s not always so self-evident...”). 

The participating students expressed a desire for earlier and better preparation for interpersonal interactions, both in dealing with patients and leading a practice team (quote: “We have (...) almost no training in team management or how to behave later in leadership roles. And also the patient contact, we’re not really prepared for that.”) 

All stakeholder groups considered social skills in dentistry essential. It was emphasized that dental treatment requires that patients feel secure during procedures. Nevertheless, many students and practicing dentists felt unprepared for dealing with people and managing a dental practice. Additionally, lecturers and students highlighted the importance of academic skills, professional attitude (e.g., work ethic, diligence), ethical awareness, and self-organization. 

### 3.2 Delphi study

Overall, the first Delphi round identified 20 relevant behaviors (out of 49) for dental *studies* (Table 2 (Tab. 2)) and 21 for dental *professional* *practice* (Table 3 (Tab. 3)). These were subsequently ranked in importance by the same group in the second Delphi round. The results are presented both as mean values across all stakeholder groups and separately by group. 

### 3.3 Evaluation of the HAM-SJT for dentistry

Across all behaviors identified in the first Delphi survey, all were at least once reflected in the items used in the HAM-SJT for medical selection processes. The most frequently recurring behaviors included “engaging with patients”, “taking patients seriously”, and “establishing transparency”.

### 3.4 Consensus rating

The results are presented in Table 4 (Tab. 4).

## 4 Discussion

Both lecturers and students in dentistry considered emotional resilience skills [36] – such as “coping with stress” and “being resilient” – to be especially important for dental *studies*, ranking among the top 5 most frequently mentioned social skills. In contrast, behaviors related to patient care, such as “being compassionate”, “taking time”, or “responding flexibly during treatment”, were regarded as less important in the context of studies, ranking 11^th^ to 13^th^. This may reflect the limited flexibility and time constraints within an intensive curriculum, as well as the limited patient contact during studies.

Conversely, four of the top five behaviors mentioned by patients and dentists concerning *practice* focus on patient interaction, including “acting in the interest of patients”, “providing a safe space”, and “taking patients seriously”. Overall, both patients and dentists assign particular importance to social engagement skills [36] such as “building trust”, “responding to patients’ needs”, and “communicating respectfully” (all within the top 10) as essential for dental practice.

All the competences and behaviors mentioned by interest-holders were already represented in the SJT developed for medical student selection. However, most competences are best assessed through a so-called Multiple Mini Interview (MMI), as it allows direct observation of concrete behaviors. While MMIs are well-known and widely used in medicine, they are comparatively cost-intensive and complex to administer, especially with a large number of applicants in the selection process. Questionnaires, such as personality inventories, can cover some of these skills but are more susceptible to bias in the selection context and are thus more suitable for self-selection.

## 5 Summary and conclusion

All experts involved in the project (from the treatment side) emphasized the importance of being prepared for interpersonal interactions, both during studies and in professional practice. Similarly, patients confirmed that the social skills of their providers are very important to them. Therefore, our findings support the incorporation of these skills into selection processes and educational curricula.

Fundamentally, the competences are quite similar to those in human medicine, with all scenarios from the human medicine SJT being linked to competences and behaviors relevant to dentistry. Since the competences and behaviors deemed important are included in the SJT used for medical selection, it is justifiable to use this SJT for dental candidate assessment as well. In principle, scenarios from both contexts could be developed for either field. However, to appropriately assess complex and nuanced skills, role-plays within MMIs are more suitable, as behavior can be directly observed. SJTs may be especially appropriate for postgraduate selection, where certain knowledge elements can already be assumed or where the goal is to identify candidates at the lower end of performance level.

For dental *studies* and *practice*, different social skills are relevant and should be considered in the selection process. Particularly during studies, students must be able to cope with stressful situations – an ability that should be assessed through alternative methods rather than SJTs. In dental practice, the competence “providing a safe space” is of central importance; it is currently only addressed indirectly in the NKLZ learning objectives under “patient-centered attitude” [1]. All relevant behaviors suitable for student selection could be assessed via MMIs, with the exception of those measurable solely through questionnaires or self-assessments. The latter methods are more prone to bias and thus less suitable for competitive selection but are more appropriate for self-selection.

## 6 Outlook and further research questions

To obtain a well-founded assessment regarding the practical implementation of “social skills in the dental curriculum”, a coordination with interest-holders in dentistry at UKE is planned. It is particularly important to gather interest-holders’ perspectives on the following questions:

1. To what extent do interest-holders believe that the described results are already addressed in the curriculum of the new iMED DENT program?
2. Do interest-holders perceive a problem with students who do not sufficiently benefit from teaching in the area of social skills? Data from communication courses so far are not available to answer this question.
3. From the interest-holders’ perspective, is the instruction of social skills during studies sufficient, or should applicants already be selected based on their social competence?
4. Regarding the finding that dental students consider “coping with stressful situations” particularly important (in contrast to practicing dentists): Do interest-holders think that especially stress-resistant applicants should be selected?

Concerning the stress experiences during studies, the following research questions arise:

1. Which factors lead to increased stress among students in dental studies (interview study)?

On the other hand, patients indicate that a dentist’s social skill of “providing a safe space” is particularly relevant for them. This suggests the potential research question:

1. How can the social skill “providing a safe space” be implemented, learned, or taught (interview study)?

The present research results support the integration of the topic “social skills” into the dental curriculum. However, the term “competence” still inherently raises the question of whether it refers to state or trait variables. Based on the current findings, the following research questions emerge:

1. Which of the competences relevant to dental study and practice are trainable to what extent, and which should already be present at the start of studies at which level?
2. Which competences relevant to dentistry are favored by the learning and working environment, and to what extent?

The implications derived from these questions for teaching and, ultimately, student selection could further advance the training of “socially competent” dentists.

## Notes

### Funding

This project was funded under the grant number NWF-20/05 by the Research Promotion Fund of the Medical Faculty of the University of Hamburg.

### Acknowledgments

We thank all interview partners and Delphi study participants for sharing their expertise with us. We also thank Ina Mielke for her support in evaluating the SJT scenarios.

### Competing interests

The authors declare that they have no competing interests.

## Figures and Tables

**Table 1:**
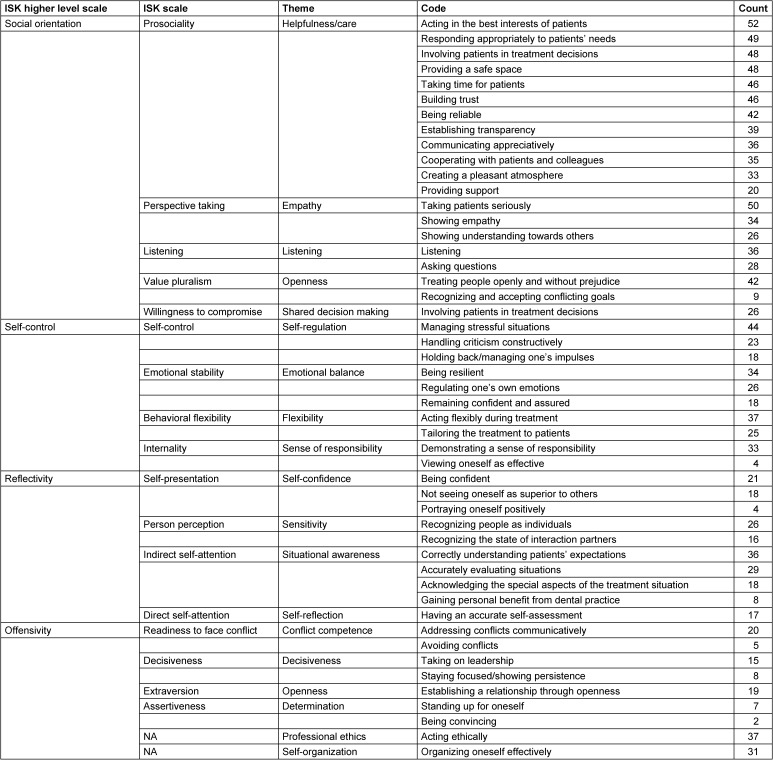
Overview of the codes, topics, and categorization within ISK scales

**Table 2:**
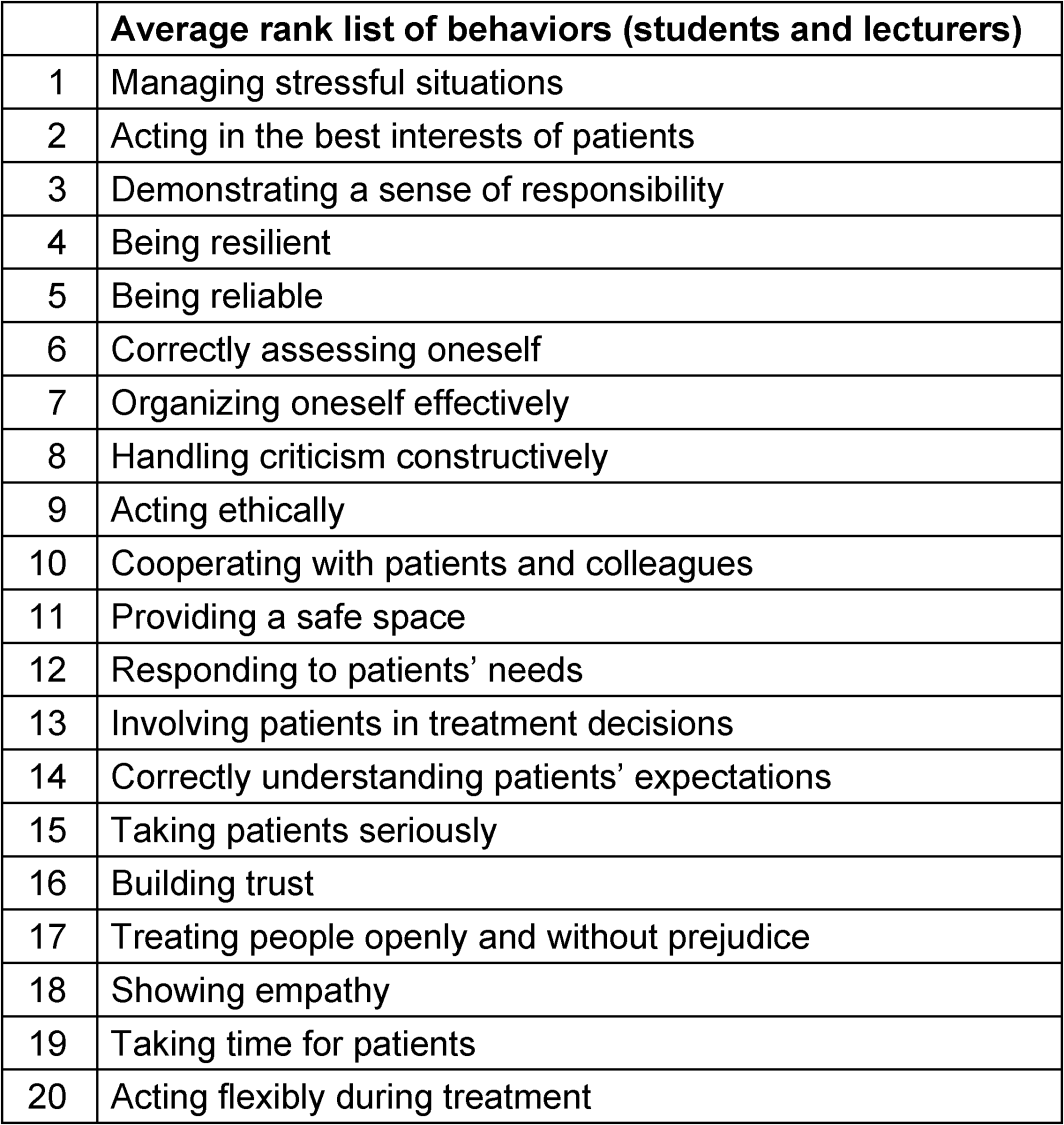
Ranking of competences and behaviors by importance for the *study* *phase*

**Table 3:**
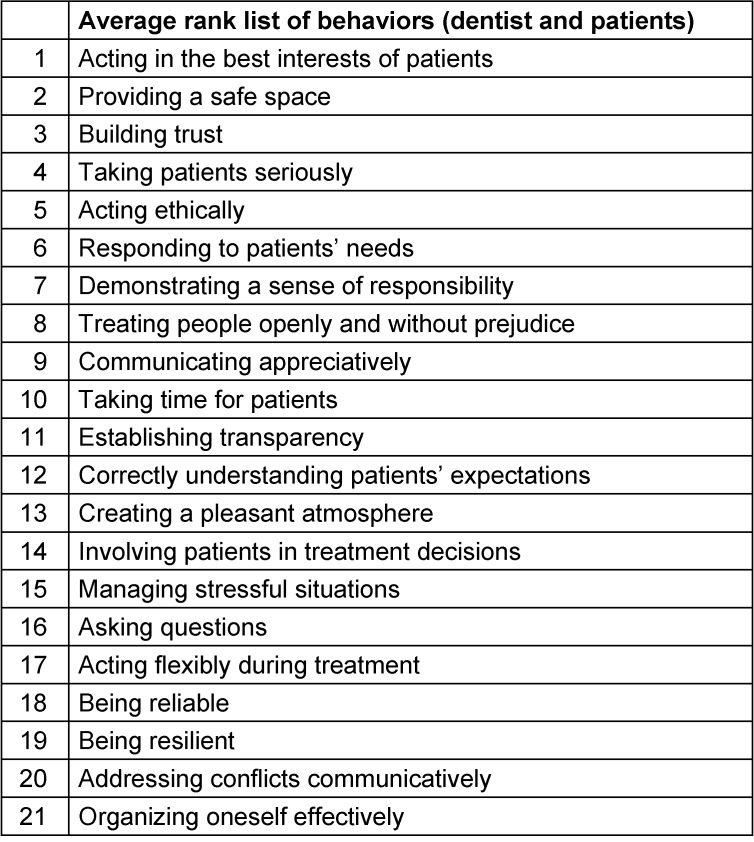
Ranking of competences and behaviors by importance for the *dental* *practice*

**Table 4:**
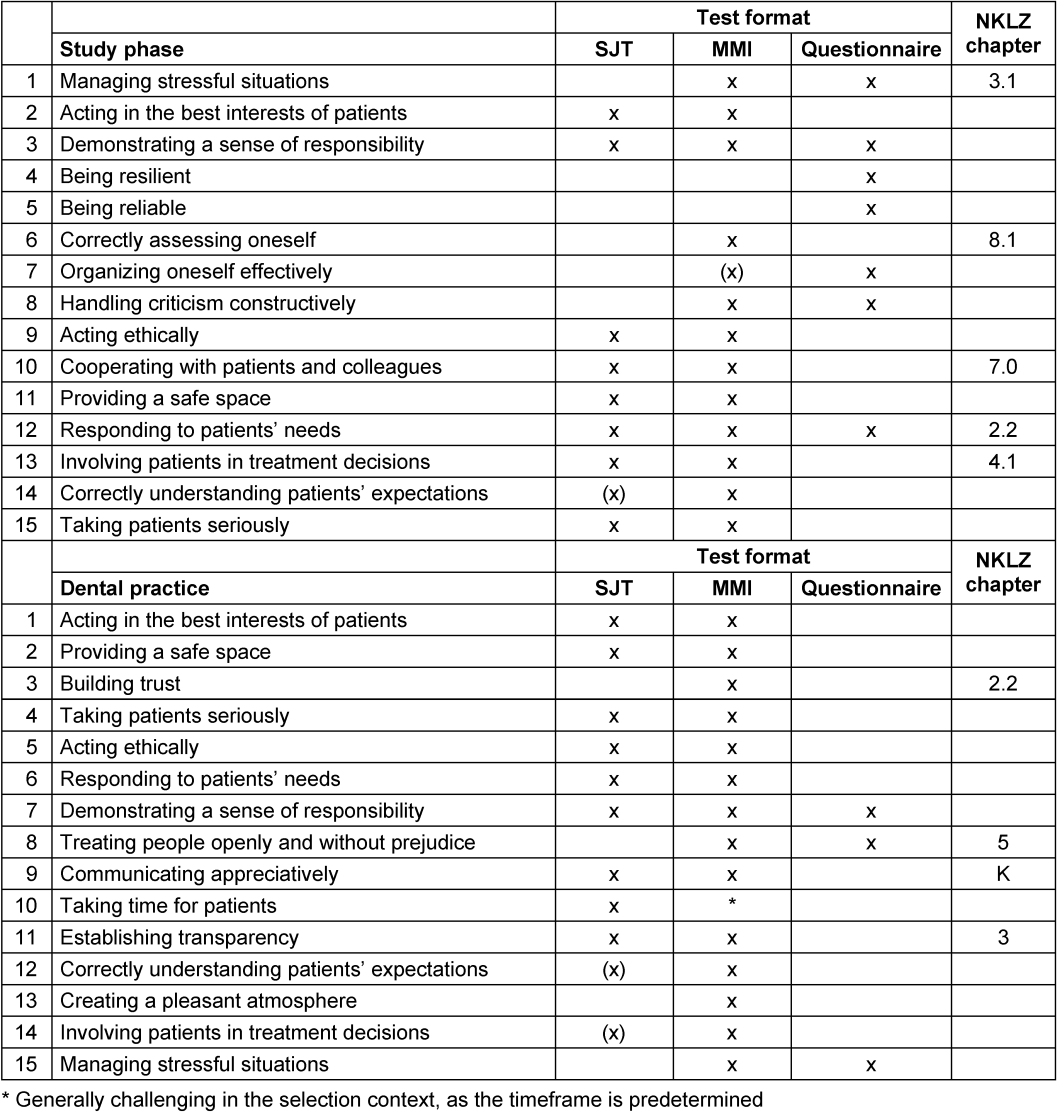
Suitability of competences and behaviors for selection/inclusion in the National Learning Objectives Catalog (NKLZ)
